# Baseline LV ejection fraction by cardiac magnetic resonance and 2D echocardiography after ST-elevation myocardial infarction – influence of infarct location and prognostic impact

**DOI:** 10.1007/s00330-019-06316-3

**Published:** 2019-08-19

**Authors:** Johannes P. Schwaiger, Sebastian J. Reinstadler, Christina Tiller, Magdalena Holzknecht, Martin Reindl, Agnes Mayr, Ivo Graziadei, Silvana Müller, Bernhard Metzler, Gert Klug

**Affiliations:** 1 Department of Internal Medicine, Academic Teaching Hospital Hall in Tirol, Innsbruck, Austria; 2 University Clinic of Internal Medicine III, Cardiology and Angiology, Medical University of Innsbruck, Anichstrasse 35, 6020 Innsbruck, Austria; 3 Department of Radiology, Medical University of Innsbruck, Innsbruck, Austria

**Keywords:** Anterior wall myocardial infarction, Magnetic resonance imaging, Cine, Echocardiography, ST-elevation myocardial infarction

## Abstract

**Objectives:**

The comparability of left ventricular ejection fraction (LVEF) measurements by cardiac magnetic resonance (CMR) and 2D echocardiography (2DE) early after ST-elevation myocardial infarction (STEMI) remains unclear.

**Methods:**

In this study, LVEF measured by CMR and 2DE (Simpson’s method) were compared in 221 patients after STEMI treated by primary percutaneous coronary intervention. 2DE image quality was systematically assessed and studies reported by an accredited examiner. Intermodality agreement was assessed by the Bland–Altman method. Major adverse cardiac events (MACE) were defined as the composite of death, myocardial infarction or hospitalisation for heart failure. Patients were followed up for a median of 40.9 months (IQR 28.1–56).

**Results:**

After non-anterior STEMI, LVEF measurements by 2DE (single and biplane) were consistently underestimated in comparison to CMR (CMR 55.7 ± 9.5% vs. 2DE-4CV 49 ± 8.2% (*p* = 0.06), 2DE-2CV 52 ± 8% (*p* < 0.001), 2DE-biplane 53.5 ± 7.1% (*p* = 0.01)). After anterior STEMI, there was no significant difference in LVEF measurements by 2DE and CMR with acceptable limits of agreement (CMR 49 ± 11% vs. 2DE-4CV 49 ± 8.2% (*p* = 0.8), 2DE-2CV 49 ± 9.2% (*p* = 0.9), 2DE-biplane 49.6 ± 8% (*p* = 0.5)). In total, 15% of patients experienced a MACE during follow-up. In multivariate Cox regression analysis, reduced LVEF (< 52%) as assessed by either 2DE or CMR was predictive of MACE (2DE HR = 2.57 (95% CI 1.1–6.2), *p* = 0.036; CMR HR = 2.51 (95% CI 1.1–5.7), *p* = 0.028).

**Conclusions:**

At baseline after non-anterior STEMI, 2D echocardiography significantly underestimated LVEF in comparison to CMR, whereas after anterior infarction, measurements were within acceptable limits of agreement. Both imaging modalities offered similar prognostic values when a reduced LVEF < 52% was applied.

**Key Points:**

*• After non-anterior STEMI, 2D-echocardiography significantly underestimated LVEF compared with cardiac MRI*

*• An ejection fraction of < 52% in the acute post-infarct period by both 2D echocardiography and CMR offered similar prognostic values*

## Introduction

Left ventricular ejection fraction (LVEF) is of major prognostic importance in many cardiac diseases, in particular after ST-segment elevation myocardial infarction (STEMI) [1–4]. Guidelines recommend transthoracic echocardiography before discharge after STEMI to assess for infarct size and resting LV function, identifying patients at high risk for worse outcome [5]. In addition, it provides important information about diastolic function in the acute phase [6]. However, there are significant limitations due to often inaccurate discrimination of the endocardial border [7, 8] and the exam can be particularly cumbersome in acute cardiac disease due to arrhythmia, stress or hypercontractility of adjacent segments [9]. In addition, its accuracy is dependent on the examiner’s experience and training and therefore offers only moderate reproducibility [10, 11].

For these reasons, cardiac magnetic resonance (CMR) imaging has evolved into the gold-standard imaging method of systolic function [12–14]; however, few studies have compared ejection fraction measurements determined by 2DE and CMR in the immediate post-infarct period. In one small study, LVEF was poorly correlated and overestimated by 2DE [15], whereas in another, larger study, LVEF was underestimated by 2DE [16]. None of these studies has systematically assessed 2DE quality or has taken infarct location into consideration.

The aim of this study was (a) to compare LVEF measurements by CMR and 2DE at baseline after STEMI, (b) to examine influence of infarct location on 2DE LVEF measurements and (c) to assess the prognostic comparability of 2DE and CMR in the immediate post-infarct period. Particular emphasis was put on retrospective assessment of 2DE image quality; all studies were reported by an individual with the appropriate experience and accreditation.

## Methods

### Study population

The patients in this study were participants in a prospective analysis enrolling patients who underwent CMR after acute STEMI treated with primary percutaneous coronary intervention (PCI) [4, 17, 18]. Inclusion criteria were the diagnosis of first STEMI according to the redefined ESC/ACC committee criteria [19] and primary PCI within 24 h of symptom onset. Exclusion criteria were renal dysfunction with an estimated glomerular filtration rate < 30 ml/min/1.73 m^2^, Killip class > 2 at time of CMR acquisition and contraindications for CMR.

Demographic data and clinical profile of patients were acquired with the help of a standardised questionnaire during hospitalisation for STEMI. Blood samples were collected as previously reported [20]. The study complies with the Declaration of Helsinki and the local ethics committee approved the study protocol. Written informed consent was obtained from all participants.

### Echocardiography

Echocardiography was performed in routine clinical practice in the Echocardiography Department of the University Hospital Innsbruck according to current guidelines [21]. Native exams were reloaded from the PACS and analysed twice by a single consultant cardiologist (JPS) at a time interval of 2 months and measurements averaged. Measurements were used to calculate intraobserver variabilities. JPS has significant experience and is certified in transthoracic and transoesophageal echocardiography by the European Society of Cardiology. JPS was blinded to CMR and clinical results. Echocardiograms were analysed on the IMAGE-COM® Software of TOMTEC Imaging Systems.

Determination of end-diastolic and end-systolic volumes was performed on four-chamber view (4CV) and two-chamber view (2CV) according to the Simpson method. Similar to others, we have used a 5-point scale to assess image quality [22]. Each 4CV and 2CV was assessed separately to increase accuracy of quality assessment. Therefore, a maximum of 10 points was allowed if there was complete endocardial definition and typical configuration (e.g. no foreshortening in 4CV or posterolateral papillary muscle visible in 2CV) in both views. Four points were allowed for each view when picture quality was mildly reduced, 3 when moderate and 2 when moderately reduced and 1 point if image quality was of borderline quality. Interobserver variabilities were calculated using 50 subjects which were selected evenly across the spectrum of 2D echocardiography quality by a second observer accredited in transthoracic and transoesophageal echocardiography by the European Society of Cardiology (GK). Observer variability analyses were performed without knowledge of patient identity or previous results.

### CMR protocol and image evaluation

All scans were performed on a 1.5-Tesla Magnetom AVANTO scanner (Siemens). Briefly, cine CMR images in short-axis were acquired using breath-hold, retrospective ECG-triggered TrueFISP bright-blood sequences. Evaluation of images was performed using a standard software (ARGUS, Siemens). End-diastolic and end-systolic volumes as well as stroke volume and LVEF were obtained from manual delineation of the endocardial borders. The most basal slice in end-systolic views was discarded if myocardium was not present in less than a half of the ventricular circumference. Trabeculation and papillary muscles were included into the LV volume. Late gadolinium enhancement (LGE) images were acquired by using an ECG-triggered phase-sensitive inversion recovery single-shot TrueFISP sequence with consecutive short-axis slices as described in detail previously [23–26]. Infarct localisation was assessed LGE CMR. Cardiac magnetic resonance images were acquired under the supervision of a EuroCMR level II–certified radiologist (AM) with a long experience in CMR (> 10 years). Analysis of CMR cine volumetric data was performed by individuals trained in volumetric dataset analysis and reviewed by at least level I–certified individuals with a long experience in CMR (> 10 years GK, AM; > 5 years SJR).

### Clinical follow-up

A LVEF of < 52% was chosen as the cut-off for reduced LVEF in both image modalities because it represented both the mean and median in the 2DE population and it corresponds to the lower limit of normal in current echocardiography guidelines for male patients [21], which fits the male predominance in our study. To ensure intermodality comparability, the same cut-off was chosen for CMR.

Patients were followed for major adverse cardiac events (MACE), defined as a composite of death, myocardial re-infarction and new congestive heart failure as previously described [27]. Time to MACE was defined as time from PCI to the first end-point.

### Statistical analysis

Statistical analysis was performed using SPSS for Windows, release 22.0.0.1 (IBM). The Shapiro–Wilk test was used to test for normal distribution. Normally distributed continuous data are presented as mean ± standard deviation (SD) and comparisons were performed by using paired *t* test. Non-normally distributed continuous data are presented as median with interquartile range (IQR) and comparisons were performed with non-parametric Wilkoxon signed-rank test. Comparisons of normally distributed continuous data in patients with or without MACE were performed using unpaired *t* test or Mann-Whitney *U* Test in the case of non-normally distributed data. Correlation of variables was performed using Pearson correlation coefficient. *P* values < 0.05 were considered to indicate statistical significance. Intermodality agreement was studied using the Bland–Altman method, whereby the mean difference was presented as the bias and 95% limits of agreement around the bias expressed as the mean difference ± 1.96 SDs. Interobserver and intraobserver variations of LVEF by 2DE were computed as the root of the mean squared differences between corresponding observations, divided by the number of observations. Intra- and interobserver correlations were calculated using intraclass correlation coefficient.

Kaplan–Meier survival curves were used to describe the cumulative incidence of event-free survival over time, and log-rank test was used to test for differences. Multivariate Cox regression analysis, adjusted for sex and age, was applied to evaluate the effect of LVEF on MACE.

## Results

### Study population

During the study period, 323 STEMI patients were enrolled into the CMR database. In 271 of these, transthoracic echocardiograms were performed in addition to CMR and pictures archived in the hospital’s PACS. In 221 of these, appropriate 4CV and 2CV were available and analysed. One patient was excluded due to severe breathing artefacts in CMR. Echocardiograms were performed a median of 3 (IQR 2–5), and CMR a median of 2.6 days after STEMI (IQR 2–4). The mean difference between exams was 0.4± 1.8 days. Mean heart rate during MR was 74 ± 16; three patients had heart rates > 110 beats/min.

The mean age of the study participants was 58 ± 11 years; 34 patients (15.4%) were female. Detailed patient characteristics on admission are shown in Table 1.

**Table 1:** Patient characteristics (*n* = 221)

Age (years)	58 ± 11
Sex (m/f)	187/34
Body mass index	26 ± 3
Diabetes (%)	13
Smoking (%)	50
Hypertension (%)	58
Total cholesterol (mg/dl)	93 ± 43
Hyperlipidaemia (%)	67%
Creatinine (mg/dl)	0.94 ± 0.2
CK max (U/l)	2110; IQR 1159–3473
Location of infarction (anterior/non-anterior)	99/122
Culprit lesion	
RCA	41%
LAD	45%
CX	13%
RI	1%
Time from symptom onset to PCI (min)	210; IQR 139–396
TIMI flow pre/post (0, I, II, III) (%)	
0	71/3.8
I	14/0.5
II	13.6/13.1
III	1.3/82.6

*CK*, creatine kinase; *IQR*, interquartile range; *RCA*, right coronary artery; *LAD*, left anterior descending artery; *CX*, circumflex artery; *RI*, ramus intermedius; *TIMI*, thrombolysis in myocardial infarction

### Echocardiography image quality

**Fig. 1:**
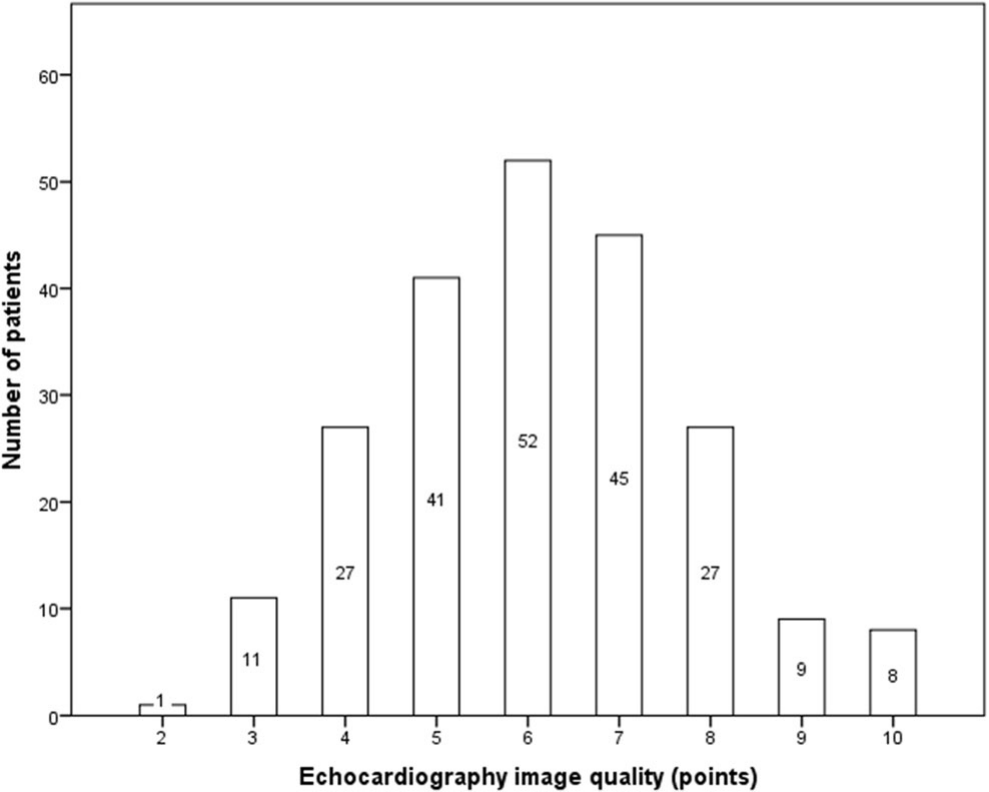
2D echocardiography image quality. Figure demonstrating even distribution of 2D echocardiography image quality. Each 4CV and 2CV was assessed on a 5-point scale allowing a maximum of 10 points. Echocardiograms of lower quality (≤ 4 points) were excluded from subsequent analysis. 4CV (four-chamber view); 2CV (two-chamber view)

Echocardiography image quality was evenly distributed, with the majority of echocardiograms being of moderate quality (5–7 points) (Fig. 1). Echocardiograms of lower quality (≤ 4 points; *n* = 39; 17.7%) were excluded, leaving 181 patients for subsequent analysis.

### Comparison of LVEF measurements and volumes

**Fig. 2:**
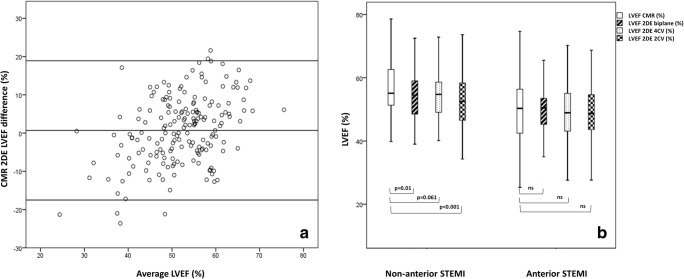
LVEF comparisons CMR and 2DE. Panel **a** Bland-Altman plot of LVEF by 2DE and CMR. Bland–Altman diagram of ejection fraction (%) demonstrating mean difference (middle line) and the limits of agreement (upper and lower lines). Panel **b** Comparison of LVEF measurements by 2DE (single and biplane) and CMR after non-anterior and anterior STEMI. Boxplot demonstrating better comparability of single- and biplane 2DE LVEF measurements with CMR measurements after anterior STEMI. Middle line in boxplot represents the median; whiskers represent 95% CI. Comparison of means performed using paired *t* test (significance level adjusted for multiplicity; *p* = 0.017). 2DE (2D echocardiography); 2CV (two-chamber view); 4CV (four-chamber view); CMR (cardiac magnetic resonance); LVEF (left ventricular ejection fraction); STEMI (ST-elevation myocardial infarction)

LVEF measurements were moderately correlated (*r* = 0.589; *p* < 0.0001) and not statistically different from each other (CMR 53 ± 11% vs. 2DE 52 ± 8%; *p* = 0.16). Only a minority of patients (12) demonstrated a severely reduced LVEF of < 35% during CMR. Figure 2 (panel a) shows the Bland–Altman plot demonstrating a small mean difference though relatively wide limits of agreement (− 17.6 to + 19%).

**Fig. 3:**
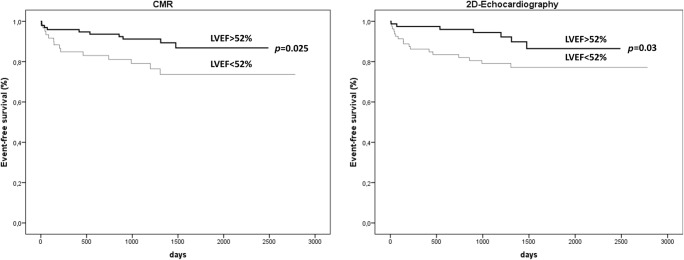
Kaplan-Meier curves for event-free survival. Kaplan-Meier curves displaying event-free survival in relation to preserved versus reduced LVEF as assessed with 2DE and CMR. CMR (cardiac magnetic resonance); LVEF (left ventricular ejection fraction)

#### Influence of infarct location

Figure 2 (panel b) demonstrates differences between LVEF measurements in anterior and non-anterior infarctions by 2DE and CMR. After anterior infarction, both single- (4CV and 2CV) and biplane LVEF measurements were not statistically different from measurements by CMR (CMR 49 ± 11% vs. 2DE-4CV 49 ± 8.2% (*p* = 0.8), 2DE-2CV 49 ± 9.2% (*p* = 0.9), 2DE-biplane 49.6 ± 8% (*p* = 0.5)).

After non-anterior infarction, all 2DE measurements underestimated LVEF (CMR 55.7 ± 9.5% vs. 2DE-biplane 53.5 ± 7.1% (*p* = 0.01); 2DE-4CV 54 ± 8.2% (*p* = 0.06); 2DE-2CV 52 ± 8% (*p* < 0.001)).

2DE significantly underestimated end-diastolic (130± 29 vs. 149± 34 ml; *p* < 0.01) and end-systolic (64 ± 20 vs. 71± 27 ml; *p* < 0.01) volumes.

#### Intra- and interobserver variabilities

The mean intraobserver difference of LVEF measured by 2DE was 1.6 ± 5.8%; mean intraobserver variability was 3.1 ± 3%. The intraclass correlation coefficient was 0.844 (KI 0.78–0.89; *p* < 0.001). Mean interobserver difference was 0.32 ± 7.9%, mean variability was 4.6 ± 3.1% and intraclass correlation coefficient was 0.801 (KI 0.65–0.88; *p* < 0.001). Intra- and interobserver variabilities of LVEF measurements by our CMR lab were recently published elsewhere [28].

### Clinical follow-up

In 161 patients (89%), follow-up was completed, and 24 patients experienced a MACE after a median follow-up of 41 months (IQR 28.1–56). In total, 40.3% of patients were classified as reduced LVEF with CMR compared with 52% when assessed with 2DE.

In univariate analysis, patients with MACE demonstrated significantly lower LVEF in both imaging modalities when compared with patients without MACE (Table 2). Figure 3 demonstrates Kaplan–Meier curves for each imaging modality showing significantly lower event-free survival in patients with reduced LVEF of < 52% (2DE *p* = 0.03; CMR *p* = 0.025; log-rank).

**Table 2:** Selected baseline characteristics in patients with and without MACE during follow-up

	MACE, *n* = 24	No MACE, *n* = 138	*p* value
LVEF CMR (%)	47 ± 13	54 ± 10	**0.005**
EDV CMR (ml)	155 ± 40	147 ± 31	0.45
ESV CMR (ml)	84 ± 36	68 ± 24	0.07*
LVEF 2DE (%)	49 ± 8	52 ± 7	**0.02**
EDV 2DE (ml)	141 ± 39	128 ± 28	0.21
ESV 2DE (ml)	75 ± 28	62 ± 18	**0.02**

*p* values < 0.05 are printed in bold

*CMR*, cardiac magnetic resonance; *EDV*, end-diastolic volume; *ESV*, end-systolic volume; *LVEF*, left ventricular ejection fraction; *MACE*, major adverse cardiac event; *2DE*, 2D echocardiography

*Mann-Whitney *U* test

In multivariate Cox regression analysis, adjusted for sex and age, reduced LVEF (< 52%) as assessed by either 2DE or CMR was similarly predictive of MACE (2DE HR = 2.57 (95% CI 1.1–6.2), *p* = 0.036; CMR HR 2.51 (95% CI 1.1–5.7), *p* = 0.028).

## Discussion

The aim of the present study was (a) to compare LVEF measurements by 2D echocardiography to the gold-standard CMR at baseline after STEMI, (b) to examine the influence of infarct location on LVEF comparisons and (c) to assess the prognostic value of reduced LVEF at baseline as assessed by both imaging modalities over a follow-up period of 41 months. As 2DE largely remains an operator-dependent technique both in data acquisition and reporting, we have put particular emphasis on retrospective assessment of 2DE image quality and all studies were reported by an individual with the appropriate experience and accreditation.

Although LVEF measurements at baseline by CMR and 2DE were not statistically different, limits of agreement were relatively wide, a phenomenon demonstrated in previous studies [29]. We demonstrated however a significant influence of infarct location on LVEF measurements by 2DE. Whereas after anterior infarction both single- and biplane 2DE measurements appeared comparable with measurements by CMR, after non-anterior infarction, LVEF was significantly underestimated in both single- and biplane measurements.

Two studies are available which have performed CMR and 2DE shortly after STEMI. A large group of 278 patients was examined and followed up by Waha et al; however, a direct comparison of LVEF was not reported, although measurements by 2DE appeared to be significantly underestimated with an absolute underestimation of 7% by 2DE [16]. Nowosielski et al have examined 52 STEMI patients and have found poor correlation of LVEF measurements demonstrating a large difference in the other direction [15]. Both studies did not report on 2DE image quality or infarct location.

More data are available after a previous infarction. The largest study was performed in 150 patients 3 months after STEMI [29]. Very similar to our results, LVEF comparisons by 2DE and CMR were very close with relatively wide limits of agreement in the Bland–Altman analysis. In a smaller study by Jenkins et al, a quarter of patients with reduced systolic function had > 10% EF difference which also corresponds to our finding of relatively wide limits of agreements [22].

Again, both studies have not reported on the influence of infarct location. Our data in a large group of STEMI patients suggest that particularly after non-anterior MI caution is advised when using 2DE for LVEF measurements. It is not known if this is a phenomenon confined to the acute post-infarct period or if this could play a role after a more distant infarction, as previous studies even with more distant infarction have not looked into this.

An explanation for the influence of infarct location might be that wall motion abnormalities at the apex after anterior infarction are reflected in both single-plane views in 2DE, whereas after inferior infarction wall motion abnormalities are usually only seen in 2CV. Even more problematic is posterior myocardial infarction which is only depicted in 3CV which is not included in the Simpson method. Furthermore, wall motion abnormalities at the apex are easier to visualise due to the proximity to the ultrasound probe, whereas e.g. the lateral wall can be more difficult to visualise due to artefacts.

In terms of prognosis after STEMI, we demonstrated that both imaging modalities offered similar predictive values for MACE when a LVEF of < 52% was applied. Several studies have shown that baseline imaging indices were powerfully and independently predictive of all major outcomes when obtained with either 2DE [30, 31] or CMR [32–34]; however, it remains unclear if the predictive values of universally accepted indices (i.e. LVEF) obtained by 2DE and CMR at baseline are comparable. Waha et al have performed both modalities at baseline and showed that several CMR parameters including LVEF added incremental prognostic value above traditional outcome markers including LVEF by 2DE [16].

Echocardiography requires a thorough knowledge of anatomy and physiology as well as technical skill which can only be gained through supervised education and training in an appropriate environment [35]. It is widely used by a wide range of technicians and physicians, both younger and less experienced on one end, up to extremely experienced examiners with log-books comprising many thousand exams, which will almost certainly make a difference, despite never being formally proven. For this reason, we have put special emphasis on assessing 2DE image quality and have excluded the echocardiograms of the lowest quality. Further, the echocardiograms in this study were reported twice by a very experienced examiner with the appropriate accreditation. In terms of LV volumes, we and others reported an underestimation of about 10% of the end-diastolic volume by 2DE [36, 37]; others however have reported an underestimation of > 30% [22, 29]. It is plausible that the degree of underestimation correlates with the degree of LV remodelling after MI. The largest net difference was reported in a patient group with very significant LV remodelling [22]. This degree of LV volume underestimation by 2DE versus CMR across studies appears to preclude comparability.

### Limitations

Several limitations of our study need to be mentioned. Firstly, a significant number of echocardiograms needed to be excluded upfront in our analysis due to the lack of appropriately stored views and low imaging quality; however, strict quality control is important in an operator-dependent technique like 2D echocardiography. Early measurements of LVEF after STEMI can be misleading because of increase in contractility in uninvolved territories [38–40] and improvement in LVEF may occur in patients who are reperfused [41]. Other factors can influence measurements in the phase of acute cardiac disease, like different loading conditions, stress, arrhythmia or tachycardia. In this study, CMR and 2DE were performed very close to each other (mean 0.4 days) but not truly sequentially; therefore, haemodynamic differences between exams cannot be fully ruled out. However, mean LV function was relatively preserved in our population. Secondly, a majority of patients were receiving beta blockers (86%) and only a minority were receiving diuretic therapy (16%). These factors as well as the normal mean heart rate during CMR (74) all indicate haemodynamic stability.

Furthermore, due to the small number of patients with severely reduced LVEF, our data cannot be extrapolated to this particularly important subgroup. Pellika et al have recently compared echocardiography with CMR in the STICH Trial, a large trial including patients with previous myocardial infarction with significant left ventricular dysfunction and have found only moderate correlation of LVEF by CMR and 2DE, although in 46% of patients only single-plane echocardiography was performed [42]. Similarly, this applies to other acute cardiomyopathies with regional wall motion abnormalities or healthy probands.

We are unfortunately unable to offer data about myocardial strain in our patients, as assessed by myocardial tagging or CMR feature tracking [43]. Myocardial strain imaging is a promising tool for better quantification of global and regional left ventricular functions in a broad range of cardiovascular diseases, including myocardial infarction or myocarditis [44–46]. Several CMR methods have been described and there is growing evidence that these techniques offer robust information after STEMI [47]. However, the improvement of risk stratification beyond traditional CMR indexes such as left ventricular ejection fraction remains controversial. Recent clinical outcome studies in STEMI patients using feature tracking or displacement encoding with stimulated echoes (DENSE) CMR indicated a strong prognostic role of global longitudinal strain or circumferential strain [48–50].

Despite these factors, our data suggest that LVEF measurements by 2DE at baseline after STEMI are generally robust provided adequate image and reporting quality and the infarct location are taken into consideration.

In summary, 2D echocardiography significantly underestimated LVEF in comparison with CMR at baseline after non-anterior STEMI, whereas after anterior infarction, measurements were within acceptable limits of agreement. Despite these differences, a reduced LVEF of < 52% by both imaging modalities had similar prognostic values for MACE over a mean follow-up of 41 months. Further study is warranted to investigate if this phenomenon is confined to the acute post-infarct period as it would have significant impact on further device therapy.

## Abbreviation

2CV: Two-chamber view
2DE: 2D echocardiography
4CV: Four-chamber view
CMR: Cardiac magnetic resonance
DENSE: Displacement encoding with stimulated echoes
HR: Hazard ratio
IQR: Interquartile range
LGE: Late gadolinium enhancement
LVEF: Left ventricular ejection fraction
MACE: Major cardiac event
PCI: Percutaneous coronary intervention
SD: Standard deviation
STEMI: ST-elevation myocardial infarction
